# Supplementary material for: A Self-Powered, Skin Adhesive, and Flexible Human–Machine Interface Based on Triboelectric Nanogenerator
Source: Nanomaterials (Basel). 2024 Aug 20;14(16):1365. doi: 10.3390/nano14161365 (PMC11356898; doi:10.3390/nano14161365)
Supplement: Supplementary file 1 [file nanomaterials-14-01365-s001.zip › nanomaterials-3131404-supplementary.pdf]

Supplementary Materials

Title: A Self-Powered, Skin Adhesive and Flexible Human-Machine Interface based on triboelectric nanogenerator

Authors: Xujie Wu [a,b,c,1]; Ziyi Yang [a,b,c,1]; Yu Dong [a,b,c]; Lijing Teng [a,b,c]; Dan Li [a,b,c]; Hang Han [a,b,c]; Simian Zhu [a,b,c]; Xiaomin Sun [a,b,c]; ; Zhu Zeng [b,c,*]; Xiangyu Zeng [a,b,c,*]; Qiang Zheng [a,b,c,*]

[a] Engineering Research Center of Intelligent Materials and Advanced Medical Devices, School of Biology and Engineering, Guizhou Medical University, Guiyang, 561113, China
[b] Key Laboratory of Infectious Immune and Antibody Engineering of Guizhou Province, Engineering Research Center of Cellular Immunotherapy of Guizhou Province, School of Biology and Engineering/School of Basic Medical Sciences, Guizhou Medical University, Guiyang, 561113, China
[c] Immune Cells and Antibody Engineering Research Center of Guizhou Province, Key Laboratory of Biology and Medical Engineering, Guizhou Medical University, Guiyang, 561113, China
[*] Corresponding author.
Email address: zengzhu@gmc.edu.cn (Z. Zeng); zengxy@gmc.edu.cn (X. Zeng); Zhengqiang@gmc.edu.cn (Q. Zheng);
[1] These authors contributed equally to this work.

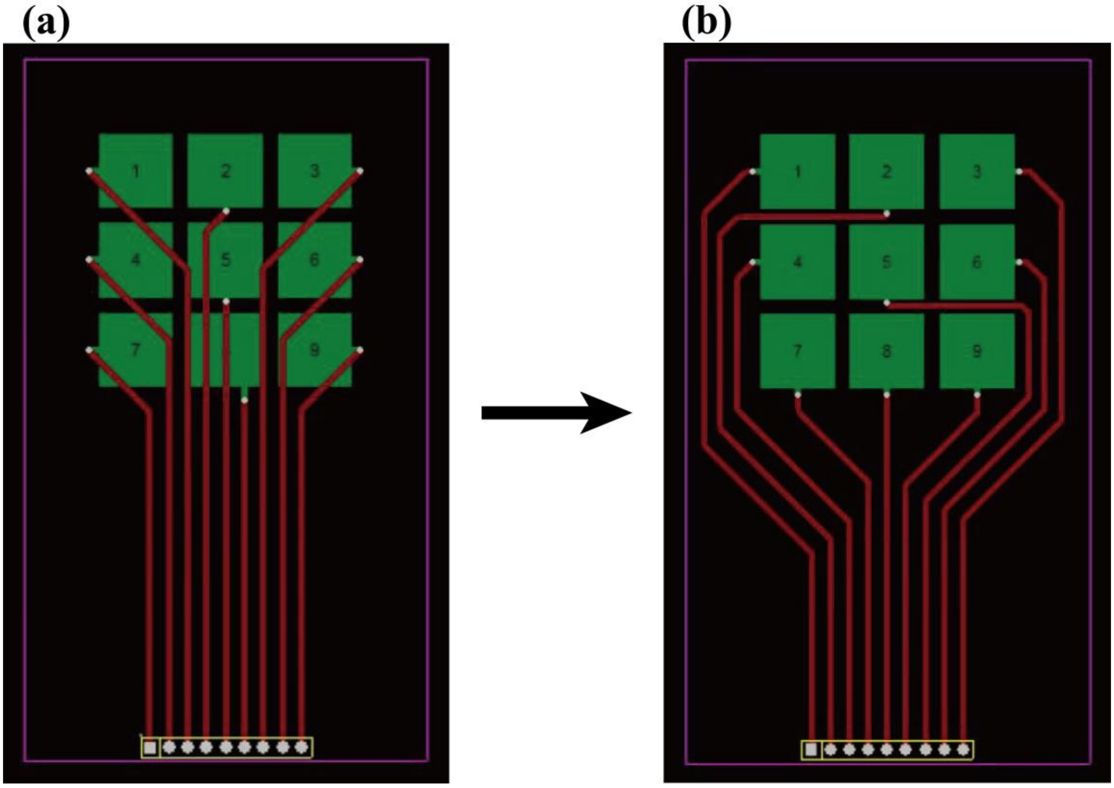

Fig. S1. The design layout drawing of the FPCB. (a) PCB routing that does not avoid 90° angles is susceptible to signal crosstalk. (b) The revised PCB routing method, coupled with supporting layers, is used to prevent signal crosstalk.

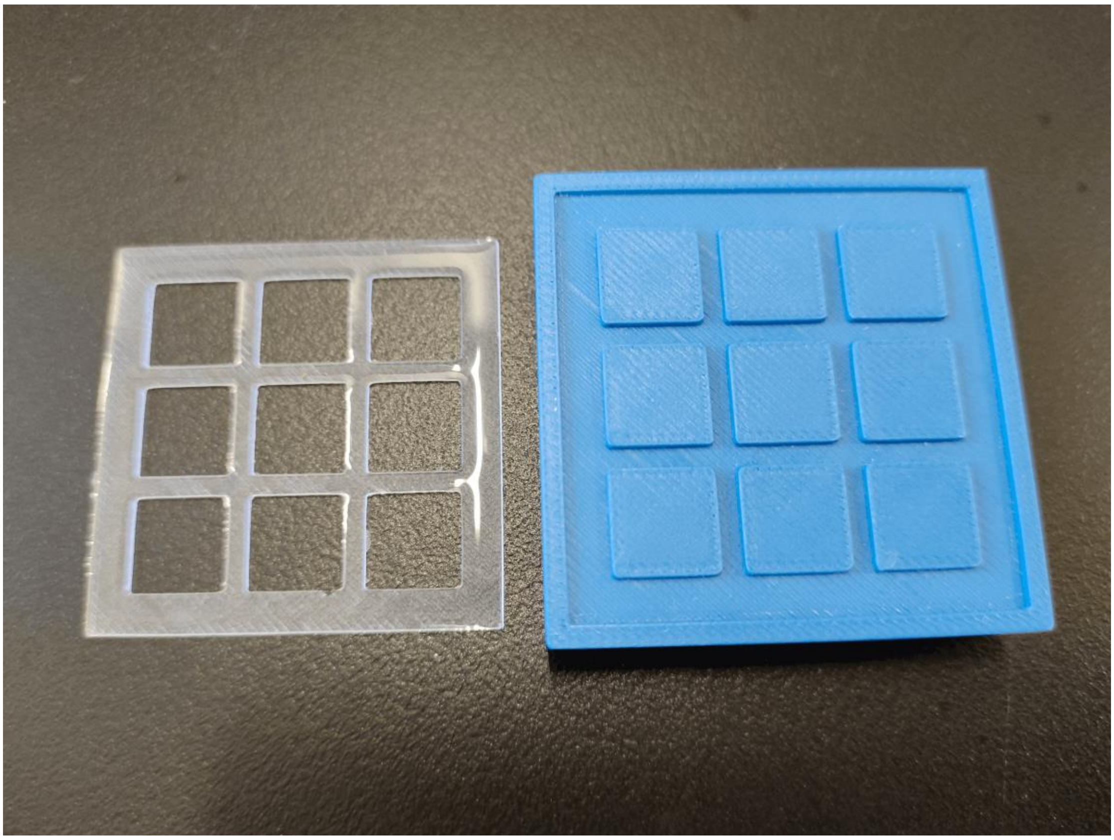

Fig. S2. The photograph of flexible PDMS skeleton and its mould.

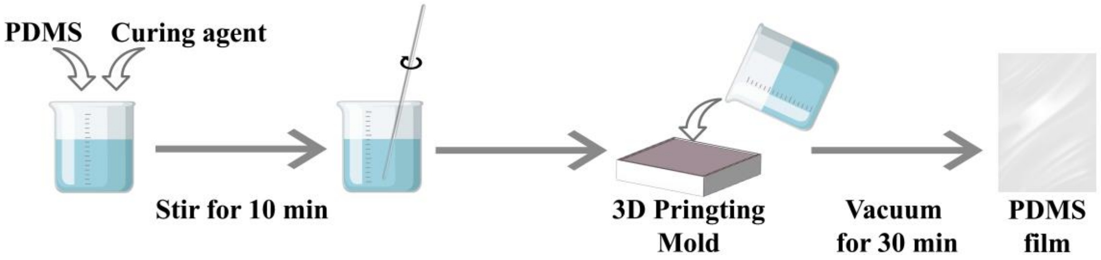

Fig. S3. The fabrication process of PDMS thin films with microstructures by using 3D printing.

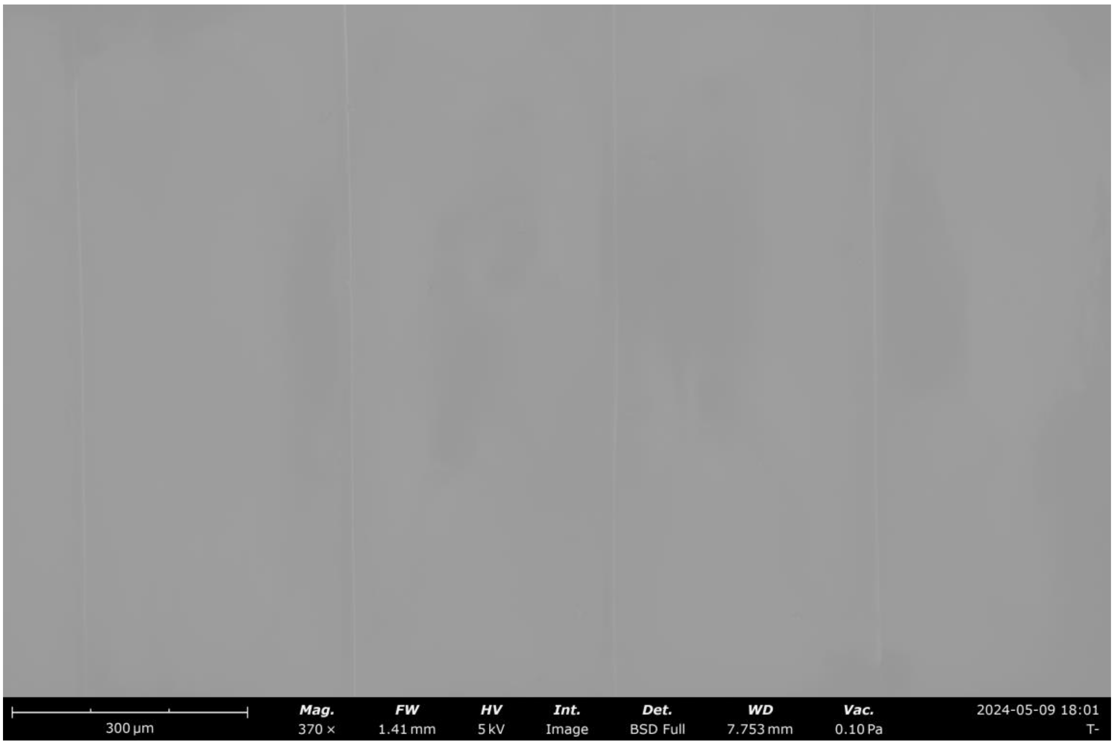

Fig. S4. The SEM images of PDMS thin films with grooves to form microstructure.

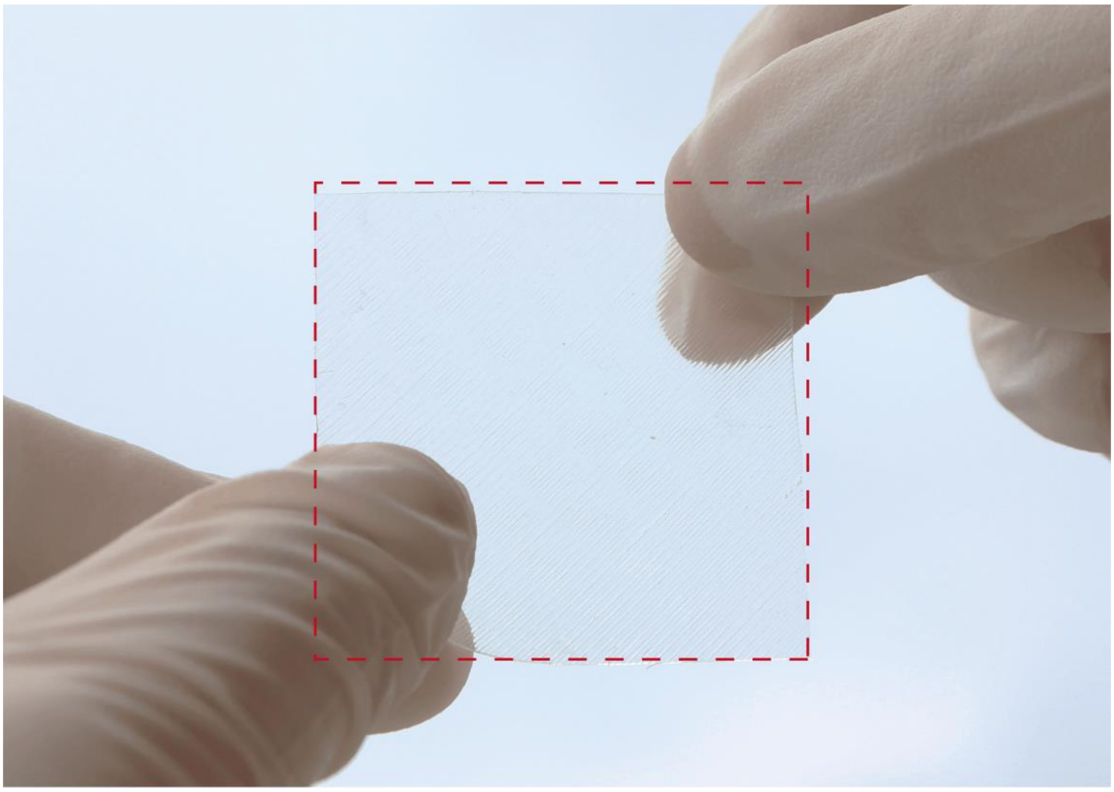

Fig. S5. The photograph of the PDMS thin film with microstructures under sunlight.

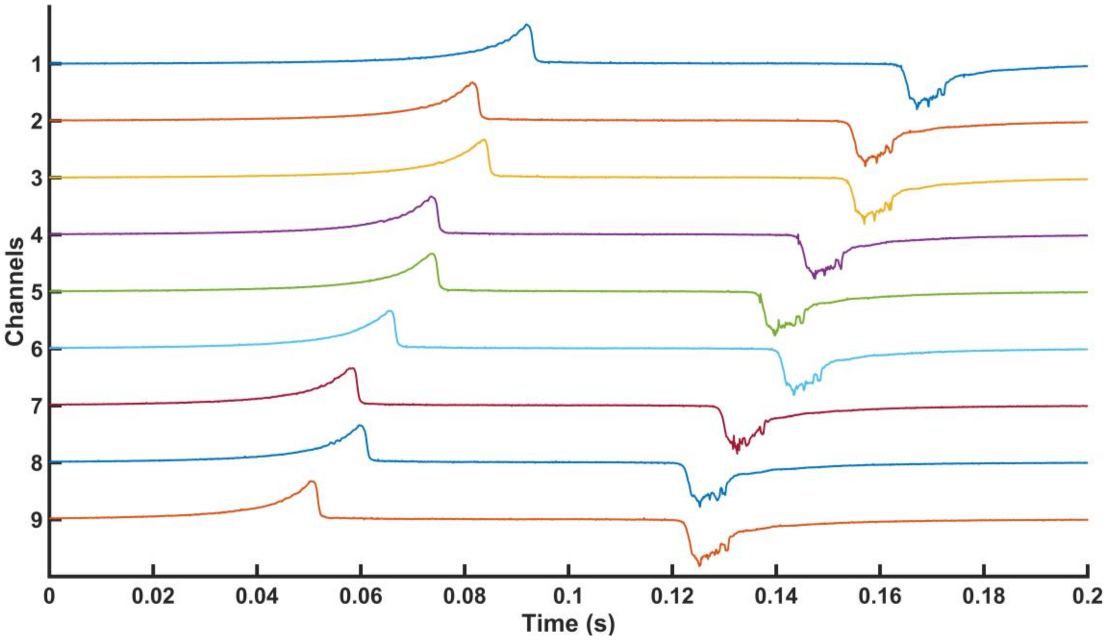

Fig. S6. The output of the TENG array with a working frequency of 5Hz, indicating the consistency of its output waveform.

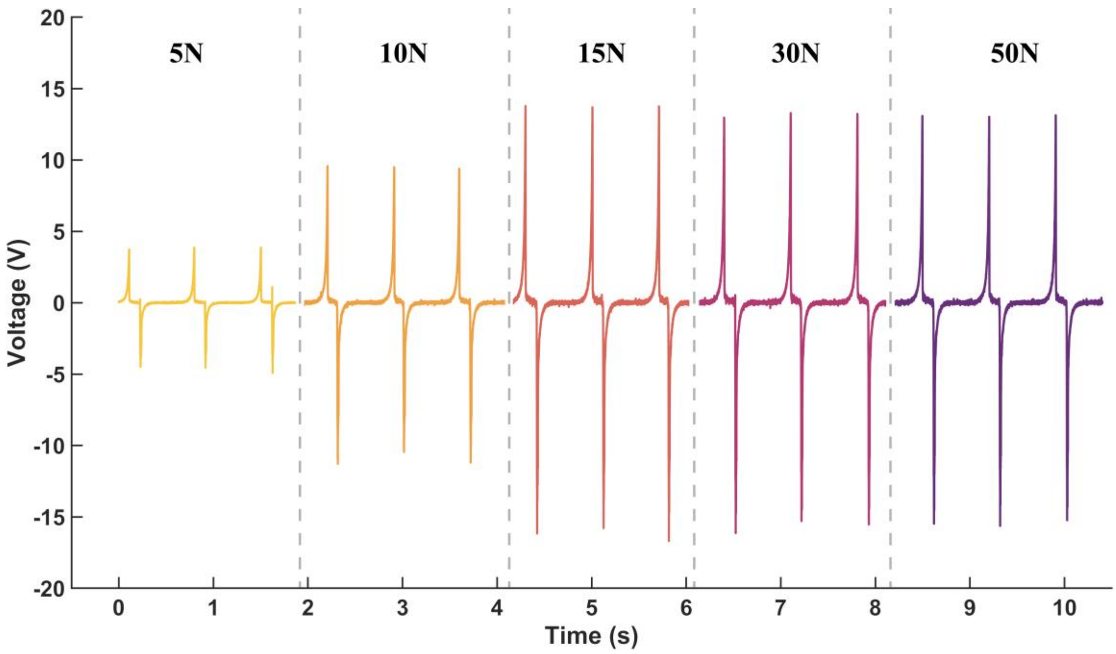

Fig. S7. Voltage response of the sensing unit on the SSFHMI to different pressures.
