# Supplementary figures and images for: A Self-Powered, Skin Adhesive, and Flexible Human–Machine Interface Based on Triboelectric Nanogenerator
Source: Nanomaterials (Basel). 2024 Aug 20;14(16):1365. doi: 10.3390/nano14161365 (PMC11356898; doi:10.3390/nano14161365)

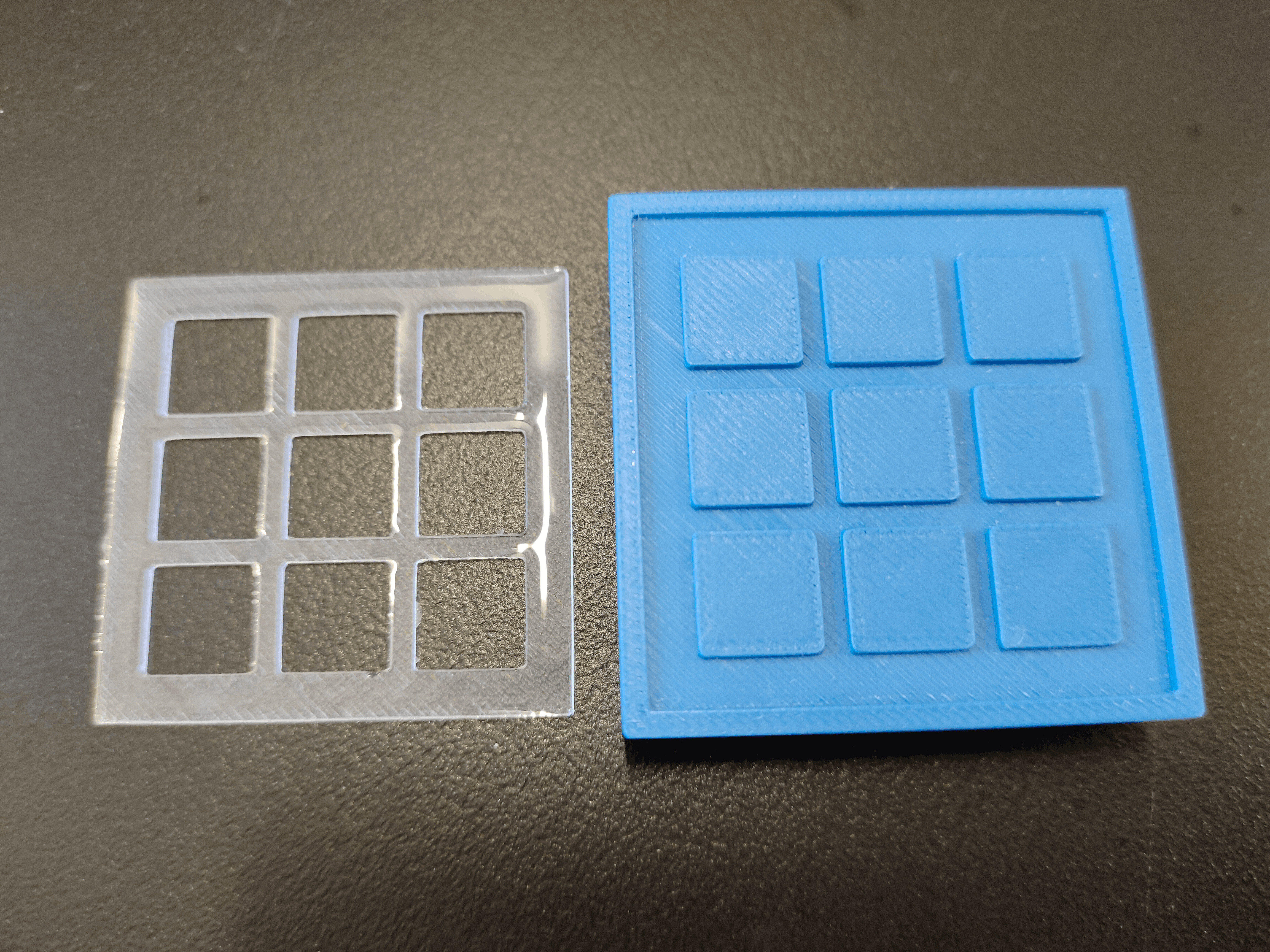

Supplement: Supplementary file 1 [file nanomaterials-14-01365-s001.zip › Figure/Fig. S2.jpg]

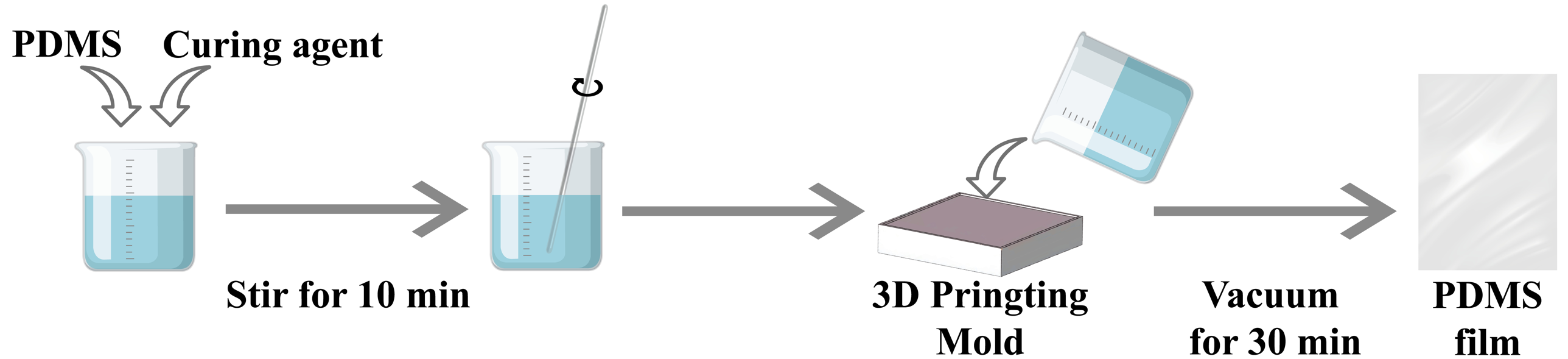

Supplement: Supplementary file 1 [file nanomaterials-14-01365-s001.zip › Figure/Fig. S3.jpg]

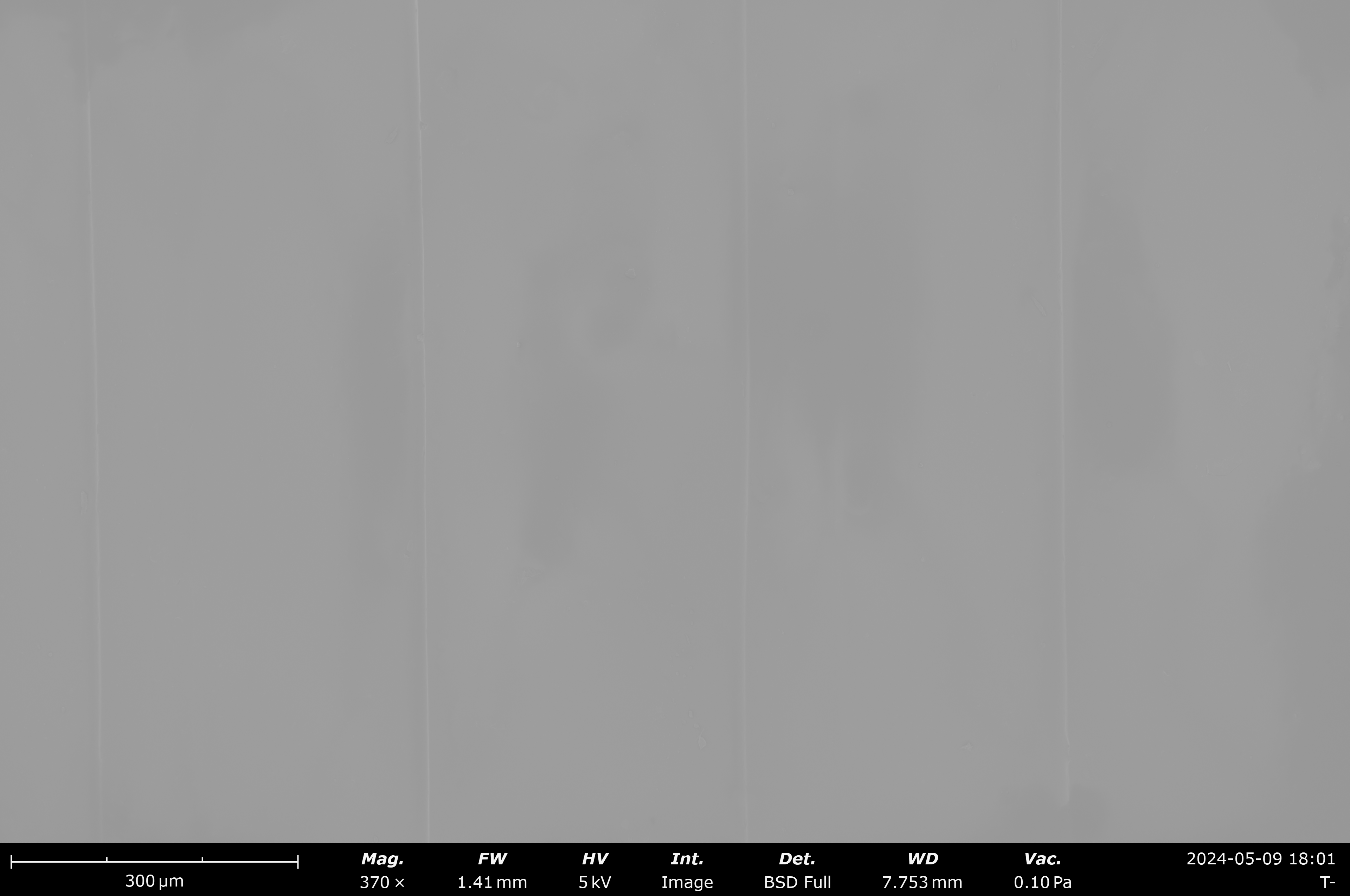

Supplement: Supplementary file 1 [file nanomaterials-14-01365-s001.zip › Figure/Fig. S4.tiff]

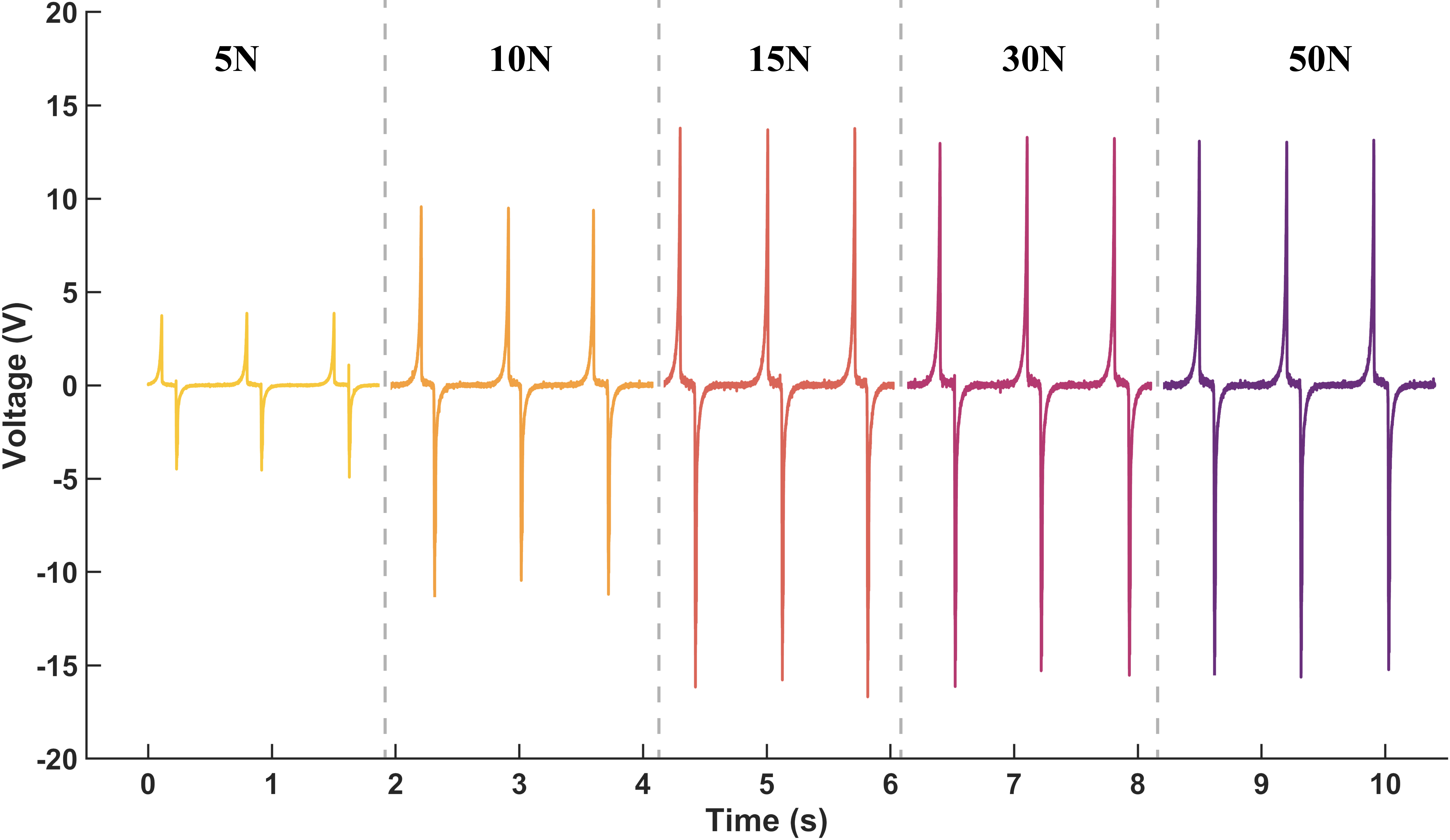

Supplement: Supplementary file 1 [file nanomaterials-14-01365-s001.zip › Figure/Fig. S7.jpg]
